# Treatment of Copper Contaminated Municipal Wastewater by Using UASB Reactor and Sand-Chemically Carbonized Rubber Wood Sawdust Column

**DOI:** 10.1155/2016/5762781

**Published:** 2016-01-20

**Authors:** Swarup Biswas, Umesh Mishra

**Affiliations:** ^1^Department of Environmental Engineering, NIT Agartala, Tripura 799046, India; ^2^Department of Civil Engineering, NIT Agartala, Tripura 799046, India

## Abstract

The performance of a laboratory scale upflow anaerobic sludge blanket (UASB) reactor and its posttreatment unit of sand-chemically carbonized rubber wood sawdust (CCRWSD) column system for the treatment of a metal contaminated municipal wastewater was investigated. Copper ion contaminated municipal wastewater was introduced to a laboratory scale UASB reactor and the effluent from UASB reactor was then followed by treatment with sand-CCRWSD column system. The laboratory scale UASB reactor and column system were observed for a period of 121 days. After the posttreatment column the average removal of monitoring parameters such as copper ion concentration (91.37%), biochemical oxygen demand (BOD_T_) (93.98%), chemical oxygen demand (COD) (95.59%), total suspended solid (TSS) (95.98%), ammonia (80.68%), nitrite (79.71%), nitrate (71.16%), phosphorous (44.77%), total coliform (TC) (99.9%), and fecal coliform (FC) (99.9%) was measured. The characterization of the chemically carbonized rubber wood sawdust was done by scanning electron microscope (SEM), X-ray fluorescence spectrum (XRF), and Fourier transforms infrared spectroscopy (FTIR). Overall the system was found to be an efficient and economical process for the treatment of copper contaminated municipal wastewater.

## 1. Introduction

The increasing population and water consumption have forced to concentrate on the reuse of wastewater. As the municipal wastewater is easy to treat and is easily available it has always been a good source for wastewater remediation. Nowadays rapid industrialization is contaminating the municipal wastewater stream by discharging toxic metal like copper which is harmful for human and other living beings. According to Environmental Protection Agency (EPA) the copper discharge limit is 1.00 mg/L. The municipal wastewater itself contains suspended solid, nutrients, and organic and inorganic pollutants which are harmful for human and environment [1, 2].

In current research trend, anaerobic treatment is observed as a good choice for wastewater treatment because it does not require oxygen which results in no energy requirement and in return it releases energy in the form of methane. As effluent generated by upflow anaerobic sludge blanket (UASB) reactor does not meet the maximum permissible level of sewage discharge standards of most developing countries including India, the posttreatment became necessary [3]. Various posttreatment options for UASB reactor effluent have been used such as anaerobic filters [4], dissolved air flotation [5], rotating biological contactor [6], overland flow process [7], down-flow hanging sponge [8], trickling filter [9], activated sludge process [10], and constructed wetlands [11]. Although most of these options have some limitations such as high operating and maintenance cost and excessive land requirement.

Slow sand filters are mainly used for the treatment of surface waters [12] as well as for the posttreatment of secondary effluents [13]. Various researchers investigated the feasibility of slow sand filters as a posttreatment unit at laboratory and pilot scale using different hydraulic loading and sand size. They suggested that slow sand filters are capable of removing biochemical oxygen demand (BOD), suspended solids (SS), turbidity, and total coliforms (TC) up to 86%, 68%, and 88% and over 99%, respectively [13, 14]. Due to its simplicity and less manpower requirement, sand filters are considered to be an economical technique.

Second convenient option for the treatment of wastewaters is the use of activated carbon (AC) as adsorbent. Adsorption provides an attractive alternative treatment because of its low cost and easy availability. AC was able to remove both organic and inorganic pollutants from wastewater [15]. In biological processes AC serves as a carrier for biofilm attachment for the treatment of wastewater [16]. Therefore, in biological activated carbon (BAC), the biological activity inside the AC system can facilitate simultaneous adsorption and biodegradation which increases the service life of AC filters [17, 18]. Due to the high adsorption and biodegradation capabilities of BAC filters, they are considered a good substitute for sand filters for removing not only suspended solids but also the organics more efficiently.

In the present study the copper contaminated municipal wastewater is treated by a laboratory scale UASB and sand-chemically carbonized rubber wood sawdust (sand-CCRWSD). The study is focused on development of a laboratory scale secondary and posttreatment system which can give better efficiency for the treatment of copper ion contaminated municipal wastewater.

## 2. Materials and Methods 

### 2.1. Preparation of Cu Contaminated Municipal Wastewater

The municipal wastewater was collected weekly at the day time from the drainages of Agartala, Tripura, India, and stored in a container. The copper contaminated (40 mg/L) municipal wastewater was prepared by adding the appropriate amount of external CuCl_2_ (Merck India) salt to municipal wastewater in another batch reactor and it was used as UASB influent. The effluent coming from the UASB reactor was treated by sand-CCRWSD column system. The UASB and sand-CCRWSD column system was monitored for a period of 121 days.

### 2.2. Preparation of the Adsorbents

The sand was collected from the nearby area of the Howrah River, Tripura, India. The sand was washed several times with deionized water and sieved for the desire particle size of 0.25–1 mm. The rubber wood sawdust was collected from rubber wood processing industry, Nagechera, Tripura, India, and it was utilized to prepare CCRWSD. Concentrated sulphuric acid (98%) and concentrated nitric acid (98%) were purchased from Merck India. The rubber wood sawdust (10 gm) was introduced to 11 mL (98% m/m) concentrated H_2_SO_4_ and stands for 10 minutes to make it carbonized. Then carbonized slurry was added to concentrated HNO_3_ (6.6 mL, 65% m/m). The slurry material was heated at 150°C for 24 hours. The CCRWSD was then thoroughly washed with deionized water to remove the acid and dried at oven. Finally screening was used to get the desired particle size (0.5–1 mm).

### 2.3. Analysis and Characterization

The copper ion concentrations in wastewater were analyzed by using the atomic absorption spectrophotometer (Perkin Elmer Model AAS 700). The surface of the adsorbent was analyzed by scanning electron microscope (SEM). X-ray fluorescence (XRF) spectrum (Model Phillips PW2404, PANAlytical) was utilized to observe the percentage of the elements present in the adsorbent. Fourier transforms infrared spectroscopy (FTIR) (Buker 3000 Hyperion, Germany) spectra were used to determine the functional groups of the adsorbent. Total phosphorus, ammonia, nitrite, nitrate, chemical oxygen demand (COD), biochemical oxygen demand (BOD_T_), total suspended solid (TSS), total coliforms (TC), and fecal coliforms (FC) of the aqueous solution were determined as per standard methods (APHA) [19].

### 2.4. Experimental Setup

**Figure 1:**
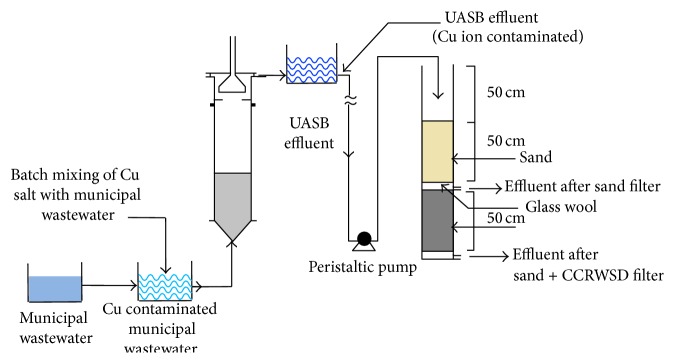
Schematic diagram of laboratory scale experimental setup.

The experimental system of the UASB and sand-CCRWSD is represented in Figure 1. The identical 34.68 L bench scale UASB reactor was run for the period of 121 days. At the batch scale UASB reactors with corresponding length, width, and depth of 17 cm, 120 cm, and 17 cm, respectively, were installed with hydraulic retention time (HRT) of 16 hrs (optimizing in different HRT). Copper contaminated municipal wastewater was used as influent for the UASB reactor. The 150 cm sand-CCRWSD column with a diameter of 5 cm was utilized as a posttreatment unit. The posttreatment column consists of two different parts where in lower portion 50 cm was filled with CCRWSD and in upper portion sand was used for 50 cm height. The 0.1 cm thick glass wool was used in between the sand column and CCRWSD column to prevent their mixing of the sand and CCRWSD. In the bottom of the column the 0.1 cm thick glass wool was used to avoid the loss of the adsorbent. The column was operated in down-flow mode at a maximum influent flow rate of 9.4 mL/min (optimizing the follow rate) at room temperature. Backwashing was made in every two weeks or when the head loss in the filter reached a critical value.

## 3. Results and Discussion

### 3.1. Characterization of the CCRWSD

**Figure 2:**
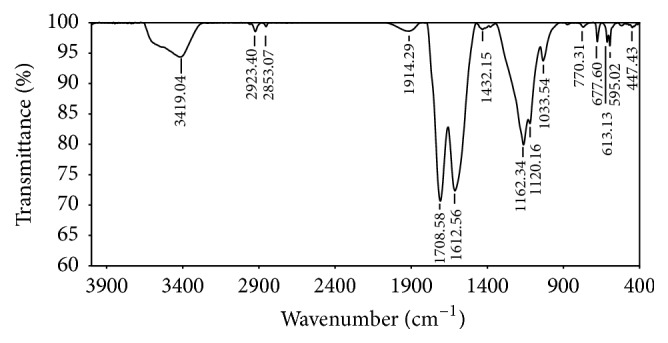
FTIR spectra of CCRWSD.

**Figure 3:**
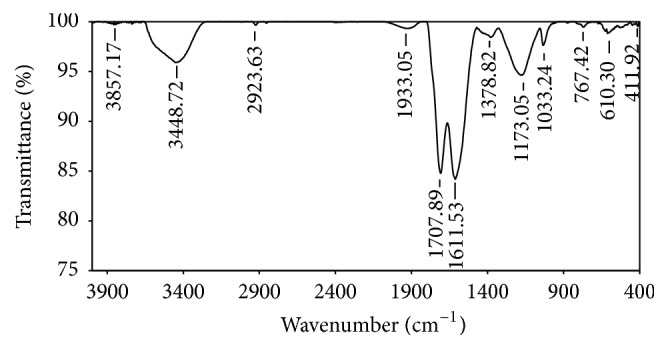
FTIR spectra of CCRWSD after filtration of UASB effluent.

**Figure 4:**
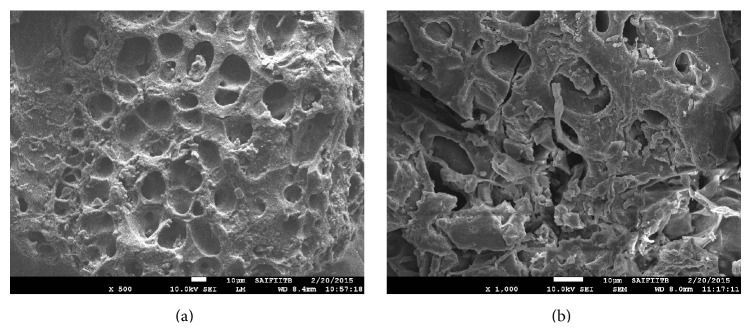
(a) SEM image of CCRWSD; (b) SEM image of CCRWSD after filtration of UASB effluent.

The functional groups of the CCRWSD are determined by using FTIR spectra which shows (Figure 2) the presence of C=O group (1708.58 cm^−1^), -COO^−^ group (1612.56 cm^−1^), and C-O group (1164.34 cm^−1^). The shifting of the peak shown in Figure 3 is due to the adsorption of the copper ion and the other impurities. SEM images show the surface structure of the CCRWSD (Figures 4(a) and 4(b)) before and after filtration of UASB effluent. The XRF analysis (Table 1) shows the presence of calcium, carbon, sulphur, and oxygen in the CCRWSD and after posttreatment the presence of the copper ion is observed which gives a strong evidence of copper ion adsorption onto CCRWSD.

### 3.2. Removal of Copper Ion

**Figure 5:**
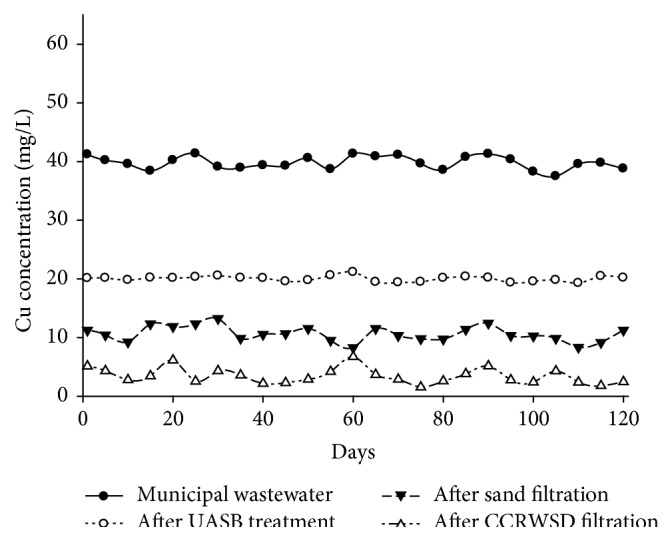
Cu ion removal.

The concentrations of copper ion after UASB and posttreatment were summarized in Table 2. It was found that copper ion concentration was gradually decreased by sand treatment and CCRWSD treatment which was shown in Figure 5. The average removal of 73.28% is found after sand treatment and after CCRWSD treatment the average removal was increased up to 91.37%. In adsorption process copper ion get defused into the hole of the adsorbents. Due to biological activation the adsorption process worked for a long time which increased the efficiency of the posttreatment unit.

### 3.3. Removal of BOD_T_

**Figure 6:**
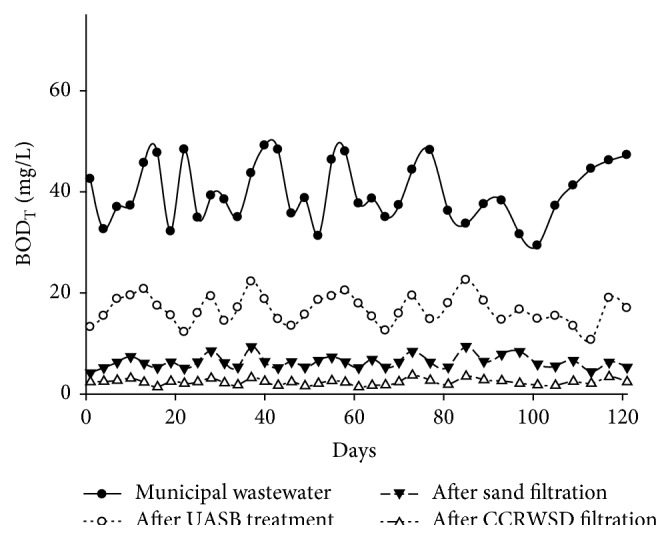
BOD_T_ removal.

The characteristics of the municipal wastewater, UASB reactor effluent, and treated wastewater were given in Table 2. The BOD_T_ of UASB reactor effluents and posttreatment effluents were decreased as compared to the untreated municipal wastewater as shown in Figure 6. The average BOD_T_ removal after UASB treatment was 83.60%. The average BOD_T_ concentration after sand filtration was 6.38 mg/L whereas after CCRWSD filter average concentration was 2.36 mg/L. Though UASB effluent had low oxygen concentration, in the upper part of the column UASB effluent came in contact with atmospheric air which helped aerobic microorganisms for the reduction of BOD_T_ in the system. Similar results were reported by Devi et al. [24] where AC, made up of Avacado Peels, was utilized for the removal of BOD from wastewater. The removal efficiency of BOD_T_ in present study is similar to the results (86%) reported by Al-Adham [25]. On the other hand BOD removal was totally dependent on the biodegradation of the organics. In primary days, BOD_T_ removal of the UASB effluent was low and increased after 2-3 days when the sand and CCRWSD became biologically active. The system was operated for a long period and for the entire period the sand and CCRWSD remain biologically active which contributed a good BOD_T_ removal efficiency.

### 3.4. Removal of COD

**Figure 7:**
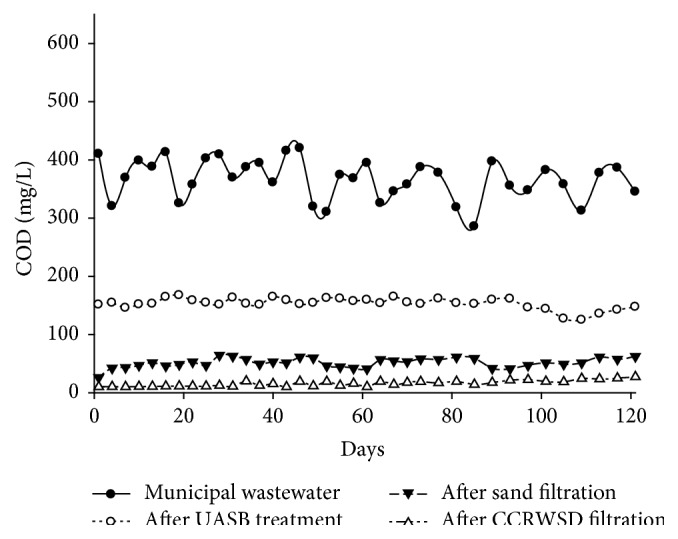
COD removal.

The COD concentration of the municipal wastewater, UASB effluent, and posttreatment effluent was summarized in Table 2. The COD was removed efficiently by the UASB treatment and the average removal of 57.82% was achieved. On the other hand, average COD concentrations after CCRWSD filtration were 15.94 mg/L with average percentage removal of 95.59% during the study period. The results showed that the UASB treatment and posttreatment column were effective for the removal of COD from municipal wastewater and were shown in Figure 7. Similar results were found by Healy et al. [26] where the performance of a stratified sand filter for the removal of COD from high-strength wastewater was studied. In literature it was observed that fly ash, brick kiln ash, and commercial activated carbons were utilized for COD remediation from domestic wastewater with removal efficiencies of 88.8% and 99% [27]. In some research works coconut coir saw dust and avocado peel carbons were also investigated for COD remediation [24, 28]. COD concentration in the UASB effluents was removed by physical, chemical, and biological processes in primary days. But after saturation, removal occurred only due to biodegradation of organics present in the UASB effluent.

### 3.5. Removal of TSS

**Figure 8:**
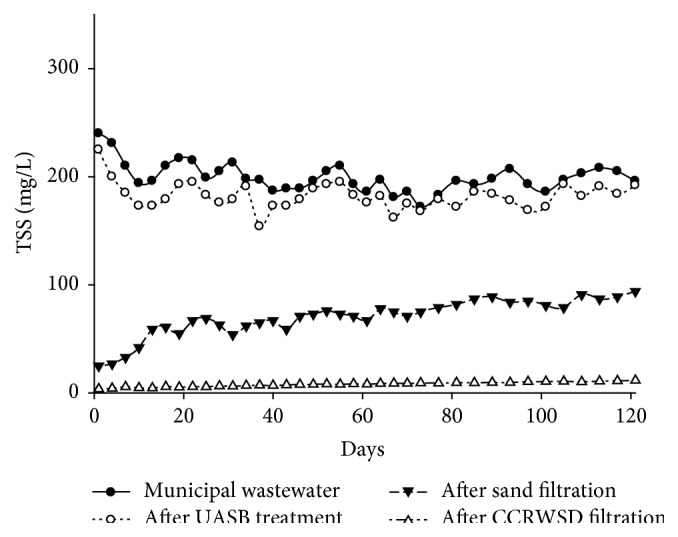
TSS removal.

The concentrations of TSS in municipal wastewater, UASB effluent, and posttreatment effluent were measured (Table 2). The removal efficiency of TSS in UASB treatment was low (average removal 8.63%) but in posttreatment unit 64.84% (average) removal was found after sand filtration and 95.98% (average) removal was achieved after CCRWSD filtration. Though the UASB treatment was not able to remove TSS effectively the posttreatment played a vital role to remove the TSS (Figure 8). The results agreed with the findings by Healy et al. [26] where 99% removal of TSS was found by using sand filter. In the study the maximum TSS removal by sand filtration was significantly higher than the results reported by Al-Adham [25], that is, only 68% at a hydraulic loading of 0.16 m/h and an effective sand size of 0.23 mm. Present observation was similar to the results by Ellis [13] as he observed 90% removal of suspended solids.

### 3.6. Nitrogen Removal

**Figure 9:**
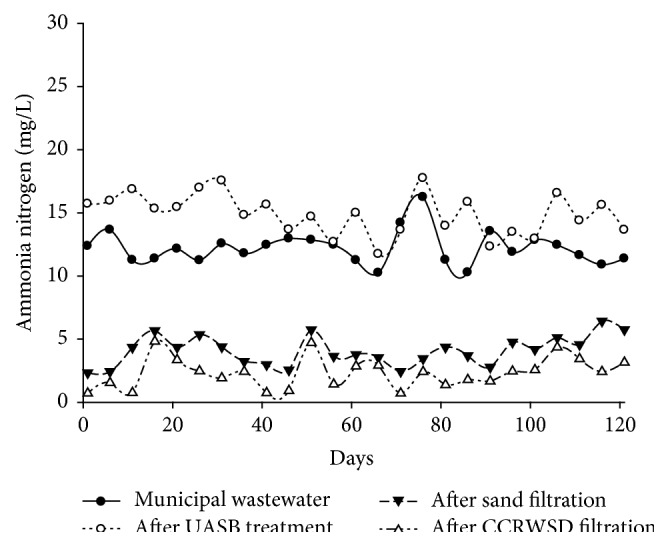
Ammonia nitrogen removal.

The concentrations of ammonia, nitrite, and nitrate in municipal wastewater, UASB effluent, and their posttreatment unit were explained in Table 2. NH_4_-N concentrations in municipal wastewater, UASB effluent, and posttreatment effluent were shown as a function of time in Figure 9. As the methane was generated in the anaerobic process the concentration of NH_4_-N was increased after UASB treatment. The average concentration of NH_4_-N in UASB effluent was 14.89 mg/L which was reduced by 65.85% by sand filtration in posttreatment unit. Similarly 80.68% average removal was found after CCRWSD column during 121 days of operation.

**Figure 10:**
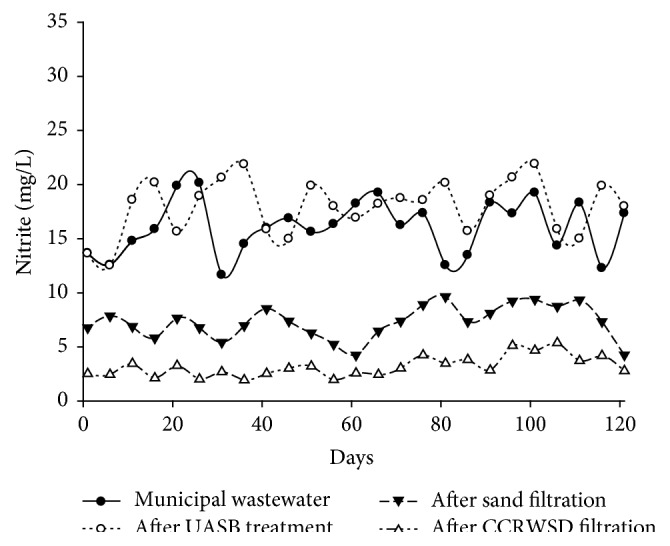
Nitrite nitrogen removal.

**Figure 11:**
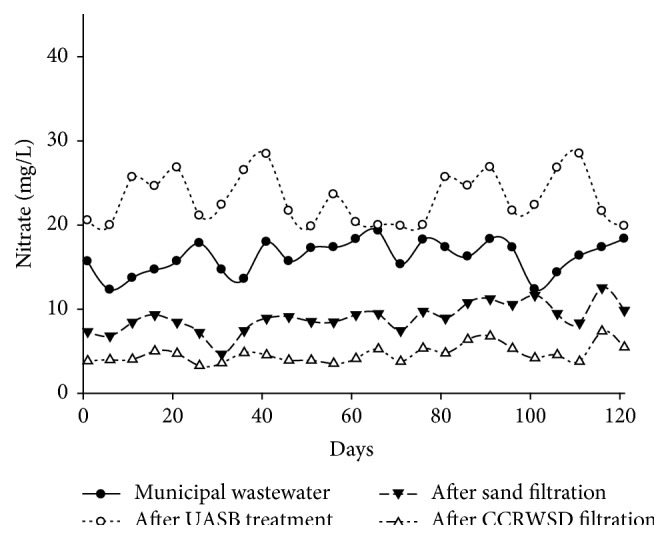
Nitrate nitrogen removal.

Simultaneously the concentrations of the nitrite and nitrate in the municipal wastewater were varied from 11.64 mg/L to 20.14 mg/L and from 12.3 mg/L to 18.36 mg/L which were also increased after UASB treatment. The effect of UASB and posttreatment unit for removal of nitrite and nitrate from municipal wastewater was shown in Figures 10 and 11. In case of nitrite average removal was 53.57% after sand filtration while after CCRWSD filtration average removal percent was 79.71%. The average nitrate removal was 44.17% after sand filtration part and 71.16% after CCRWSD filtration. During this period experiments revealed that oxygen concentration in UASB effluent was always less and therefore the environment was suitable for denitrification. In one of the previous studies [18] the authors observed 95% nitrification efficiency in BAC columns. Lower nitrification efficiency is observed in the current study due to low dissolved oxygen concentrations. Higher NH_4_-N concentrations in the secondary effluent demand higher oxygen consumption for biodegradation. Organic nitrogen mostly remains in the form of NH_4_ which usually oxidize to NO_2_ and finally to a more stable form of nitrogen (NO_3_). Nitrogen concentrations were effectively removed in the sand-CCRWSD system by nitrification and denitrification process. The autotrophic and heterotrophic bacteria present in the UASB effluent started nitrification from the first days of operation and reduced ammonia. Low oxygen concentration is good for denitrification [18] in UASB effluents. Therefore, biologically activated sand-CCRWSD system removed total nitrogen concentration in the form of ammonia, nitrite, and nitrate efficiently for the long period of operation.

### 3.7. Phosphate Removal

**Figure 12:**
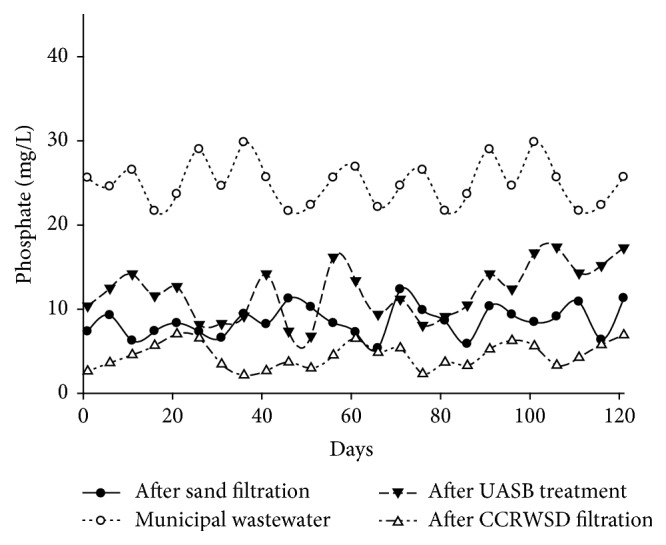
Total phosphorous removal.

The concentration of phosphate in municipal wastewater varied from 5.86 mg/L to 12.34 mg/L. In the anaerobic treatment concentration of phosphate in UASB effluent was increased (shown in Table 2). The average PO_4_ concentrations in UASB effluent and after sand filtration were 25 mg/L and 12.04 mg/L. After sand filtration the average removal of 4.5% was achieved whereas after CCRWSD filtration the average removal percentage was 44.77%. The fate of phosphorous concentration and its percent removal after UASB, sand filtration, and CCRWSD filtration with increasing days is shown in Figure 12. In one study of sand filter total phosphorous removal was 36% to 37% which is slightly similar to the present experimental results [29]. Rodgers et al. [30] stated the 90% removal of the phosphorous by sand filtration. The decrease in the PO_4_ concentration could be due to the decomposition of some organic phosphorus from the wastewater by the activated sand and the activated carbon. Microorganisms present in biologically active system use phosphorous and nitrogen for their cell growth [30, 31]. The biologically activated sand-CCRWSD system had the presence of bacteria, protozoa, algae, and various microorganisms which utilized phosphorous as nutrient and decreased total phosphorous concentration from UASB effluent.

### 3.8. Removal of TC and FC

**Figure 13:**
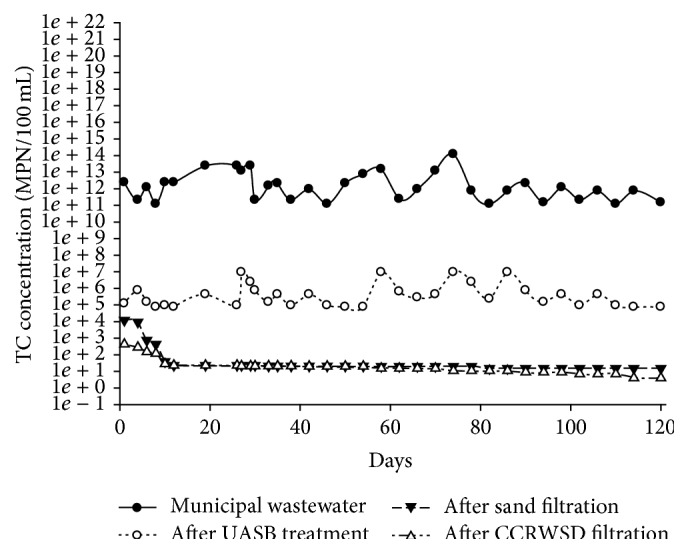
Total coliform removal.

**Figure 14:**
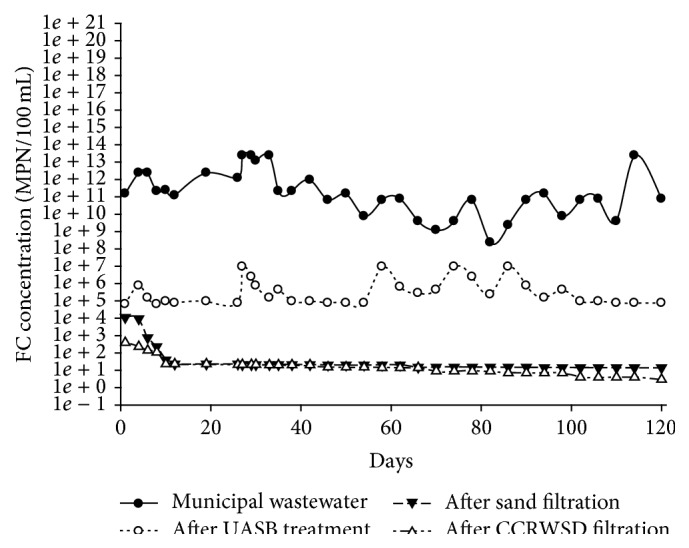
Fecal coliform removal.

The concentrations of TC and FC in municipal wastewater were described in Table 2. A very good amount of removal was taking place in UASB treatment shown in Figures 13 and 14. During the study period after sand filtration TC concentrations in the effluent were 15 MPN/100 mL to 1.2 × 10^4^ MPN/100 mL and after CCRWSD filtration they were 4 MPN/100 mL to 4.3 × 10^2^ MPN/100 mL. FC concentrations in the effluent after sand filtration and after CCRWSD filtration were 14 MPN/100 mL to 1.1 × 10^4^ MPN/100 mL and 3 MPN/100 mL to 3.9 × 10^2^ MPN/100 mL. In case of TC and FC after posttreatment 99.99% and 99.99% removal were achieved. The removal of pathogens in the present study was significantly higher than the result by Al-Adham [25] as reported in literature. Similarly Bellamy et al. [32] reported that the average coliform removal was 97% in their study. The present study concludes that in this system almost 99.9% coliform (TC and FC) removal was achieved after 10 days and TC and FC concentrations were also observed below the discharge limit as prescribed by WHO [33]. In first few days, nutrient-rich UASB effluent leads to the formation of biologically active mats which is the mixture of photosynthetic microorganism and heterotrophic bacteria referred to as “Schmutzdecke” [34]. Due to better oxygen availability the growth of biomass was high in the upper part of the filter which gave higher removal efficiency of the coliform. The maximum removal of coliforms was achieved by the sand bed and the remaining coliforms were removed by the CCRWSD.

### 3.9. Comparison of UASB + Sand-CCRWSD Column with Other Treatment Units

The comparison of UASB + sand-CCRWSD column with the other treatment unit for the removal of contaminants is illustrated in Table 3. Comparing with the other treatment technique the system is found as the efficient technique to remove the COD, BOD_T_, ammonia, nitrate, and fecal coliform. It is observed that the sand and CCRWSD system leading to best adsorption would probably be the most suitable type for biodegradation as well.

## 4. Conclusions 

This study shows that the copper contaminated municipal wastewater can be treated effectively by the combine system of UASB and sand-CCRWSD column. Simultaneously the system can efficiently remove BOD_T_, COD, TSS, nitrogen, phosphorous, and coliforms. Therefore, the UASB and sand-CCRWSD column system can lead to one of the best economical municipal wastewater treatments and can probably be considered as the most suitable type for biodegradation process.

## Figures and Tables

**Table 1:** XRF analysis on the CCRWSD before and after treatment of UASB effluent.

Elements (%)	CCRWSD	Lead-loaded CCRWSD
O	31.24	21.54
C	61.41	59.21
Na	0.020	0.002
Ca	0.427	0.14
Cu	—	2.47
S	0.643	0.081
Cl	0.190	0.078

**Table 2:** Characteristics of UASB and posttreatment unit effluents.

Parameters	Municipal wastewater	UASB effluent		After sand filtration		After CCRWSD filtration	
	Range	Range	% removal	Range	% removal	Range	% removal
Copper ion (mg/L)	37.84–41.35	19.27–21.15	47.39–52.87	8.29–13.24	66.14–79.94	1.54–6.72	83.74–96.12
COD (mg/L)	310.4–420.1	125.1–167.5	46.67–64.46	26.3–64.7	80.75–93.59	10.2–27.4	92.06–97.46
BOD_T_ (mg/L)	29.35–49.14	10.68–22.23	33.06–74.66	4.22–9.45	71.93–90.07	1.4–3.4	89.60–97.06
TSS (mg/L)	172–240	154–225	2.03–21.82	25–94	52.04–89.58	3.7–11.6	94.08–98.46
TDS (g/L)	1.86–2.01	1.70–1.91	1.59–13.06	0.35–1.89	0.48–81.57	0.32–1.87	1.40–83.45
Ammonia (mg/L)	10.24–13.65	11.73–17.74	nil	2.34–6.43	40.96–82.76	0.71–4.82	57.60–95.00
Nitrite (mg/L)	11.64–20.14	12.51–21.89	nil	4.25–9.65	23.05–75.37	1.94–5.37	65.85–89.97
Nitrate (mg/L)	12.3–18.36	19.82–28.47	nil	4.67–12.56	5.50–59.41	3.27–7.38	57.46–79.60
Phosphate (mg/L)	5.86–12.34	21.64–29.84	nil	6.8–17.4	nil	2.16–7.04	9.15–77.07
Total coliform (MPN/100 mL)	1.20 × 10^11^–1.20 × 10^14^	7.5 × 10^4^–9.3 × 10^6^	99.99	15–1.2 × 10^4^	99.99	4–4.3 × 10^2^	99.99
Fecal coliform (MPN/100 mL)	2.30 × 10^8^–2.40 × 10^13^	6.4 × 10^4^–9.3 × 10^6^	99.99	14–1.1 × 10^4^	99.99	3–3.9*E* × 10^2^	99.99

**Table 3:** Comparison of UASB + sand-CCRWSD column with other treatment units.

Technology	COD (mg/L)	BOD (mg/L)	TSS (mg/L)	NH_4_ (mg/L)	NO_3_ (mg/L)	Fecal coliform (MPN/100 mL)	References
UASB + DHS	62	16.5	17.5	8.8	6.6	3.8 × 10^4^	[10]
UASB + AFB	61.66	18	19				[14]
UASB + RBC	95			24	22.9		[6]
UASB + shallow PP		27	26				[20]
UASB + ozonation	53	20	13		0.94	84	[21]
UASB + ASP	128		99				[22]
UASB + AF	114	28	32				[4]
Inland surface water standard	250	30	100	50	50	10000	[23]
Drinking water standard		2		—	45	0	[23]
UASB + sand-CCRWSD	10.2	1.4	3.7	0.71	3.27	3	Present study
